# Does congruence between a descendant entrepreneur’s personality traits and family business values matter for succession?

**DOI:** 10.3389/fpsyg.2023.1043270

**Published:** 2023-02-09

**Authors:** Zeshan Ahmad, Wai Meng Chan, Elaine Yen Nee Oon

**Affiliations:** Faculty of Business and Economics, University of Malaya, Kuala Lumpur, Malaysia

**Keywords:** big-5 personality traits, family business value congruence, succession success, small family business, person-organization fit theory

## Abstract

**Purpose:**

In this paper, we investigate two research queries pertaining to the success of small family business succession. First, we examine how the Big-5 personality traits of descendant entrepreneurs influence the success of their family business succession. Second, we investigate whether descendant entrepreneurs whose personality traits are congruent with the values of their family business, would lead to the success of their family business succession, through the mediating role of descendant entrepreneur-family business value congruence (DE-FBVC).

**Methodology:**

We rely on the person-organization fit theory for our conceptual framework and we collected primary data from 124 respondents designated as chairman and managing directors in small family businesses.

**Results:**

Our results show that a descendant entrepreneur’s openness, extroversion, conscientiousness, and agreeableness traits are likely to lead to successful family business succession, but a descendant entrepreneur with neuroticism trait is unlikely to do so. In addition, our results reveal that the DE-FBVC mediates the relationship between openness and extroversion traits with succession success positively, but between neuroticism trait and succession success negatively. By contrast, we find that DE-FBVC does not mediate the relationship between conscientiousness and agreeableness traits with succession success.

**Originality:**

The findings of our study suggest that while four of the Big-5 personality traits matter for the success of small family business succession, specific personality traits of descendant entrepreneurs which are found to be congruent with the values of their family business, will also lead to succession success.

## 1. Introduction

One of the most intriguing questions in the literature is why some small family businesses can survive beyond one generation while others fail (Poza, 2021). Succession is a rigorous process and a complex entrepreneurial puzzle (Ahmad and Yaseen, 2018; Oliver et al., 2018). The rate of succession success for small family businesses declines as they mature (Chanchotiyan and Asavanant, 2020). Statistics reveal that almost 70% of family-controlled small family businesses find it challenging to make it through the second generation, while 90% of small family businesses cannot survive beyond the third generation (Diaz-Moriana et al., 2020). This downward trend of small family business survival rate is not only a serious threat to families in earning their bread and butter but also for the countries to maintain their employment, exports, and GDP growth rates (Schwab, 2021).

A small family business is controlled by the founder or his descendants across generations (Ahmad et al., 2018). Each descendant has different entrepreneurial, cognitive capabilities and vision to rationalize complex issues like succession (Murad et al., 2022). An entrepreneur’s attitude, behavior, and cognitive capabilities are the reflections of an individual’s personality traits (Zicat et al., 2018). Hence, every individual having different personality traits anticipate the grievousness of the matter differently and take initiative to resolve the raised issues based on his abilities.

The Big-5 personality traits have been found to have a significant influence on how entrepreneurs tackle complex matters such as entrepreneurial endeavors and the sustainability of their businesses through challenging times (Soto Maciel et al., 2015; Lu et al., 2022). Past studies have examined the influence of Big-5 personality traits of a founder or CEO of SMEs on the performance of the firms they own and manage (Antoncic et al., 2018). These studies revealed that Big-5 personality traits may play a significant role in the management and sustainability of small family businesses but are silent on the relationship between personality traits and succession matters. For example, would a descendant of an entrepreneur who takes over the control of the small family business^1^ and who is open, conscientious, extrovert or agreeable be better at continuing the family firm and legacy relative to one who has neuroticism trait? Unfortunately, literature is scant about how the Big-5 personality traits of the descendant entrepreneurs influence the success of their small family businesses successions. This motivates our first research question: *How does a descendant entrepreneur’s personality traits influence the success of small family business succession?* Our aim is to discover which personality trait or traits (within the Big-5) of a descendant entrepreneur would contribute to the success of small family business succession.

Our first research question warrants further empirical examination for three reasons. First, it is rather surprising that while personality has long been investigated in the CEO succession literature, the family business succession literature is silent on how the personalities of descendant entrepreneurs influence the succession success of family firms (Rothwell and Prescott, 2022). Evidence around the world has shown that successful succession beyond the first or second generation in family firms is quite uncommon; hence we conjecture that firm-level and external environmental issues may not be the only influential factors determining successful succession. Second, because of the strong emotional and familial ties unique to family firms, the personality traits of a descendant entrepreneur may be the potential linchpin toward the successful continuity of family firms and legacy beyond the first generation. Third, since small family firms usually have limited human capital resources to lead and manage the firm, the personality traits of a descendant entrepreneur and the congruence between the descendant entrepreneur’s personality traits and firm values become particularly important.

Family business researchers have advocated that the outcome of personality traits on business performance (i.e., succession success) should be investigated through mediating mechanism (Del Bosco et al., 2019). Personality traits of a descendant entrepreneur may improve the likelihood of succession success, but the literature is also silent on whether the descendant entrepreneur’s motivation may play a role between the descendant entrepreneur’s personality traits and succession transition (Byrne et al., 2019). An individual is motivated when his personality traits are congruent with the values of the firm (Arieli et al., 2020). Therefore, value congruence is an important mediator which should be investigated between personality traits and succession success. In addition, past studies have demonstrated congruence between a top leader’s unique profile of personality and the values of the firm. The leader nurtures the values of the organization congruent with their personality traits and tends to surround themselves with individuals who are similar to themselves to achieve the set goals of the organization (Muñiz-Velázquez and Frade, 2021). Thus, we propose that a descendant entrepreneur whose personality is congruent with the family firm’s values would be the best fit to lead the family firm into the next generation successfully.

Moreover, the mediating role of value congruence is also supported by the person-organization fit literature. This literature postulates that people tend to be attracted to and are likely to work toward achieving their firm’s profitability and longevity as well as remain with organizations in which they perceive a fit due to congruence between their traits and the organization’s values (Muñiz-Velázquez and Frade, 2021). Hence, we predict that descendant entrepreneurs of family firms whose personality traits congruent to those of their firms’ values are likely to remain with the firm, resulting in successful succession to the next generation (Motylska-Kuzma et al., 2022). Consequently, we fill a research gap by investigating the congruence between the descendant entrepreneur’s personality and organization’s values within the context of small family businesses in our second research question: *Is the descendant entrepreneur’s Big-5 personality traits congruent with the values of the small family business, thereby leading to the success of the family business succession?* We rely on the person-organization fit theory pioneered by Hsu et al. (2019) in developing our predictions on how successful succession depends on the congruence between the descendant entrepreneur’s personality traits and the values embedded within their family firm.

We test our predictions using a sample of small family businesses within the retail sector in Pakistan. Data was collected from 124 descendant entrepreneurs designated as chairman and managing directors. Our results suggest that descendant entrepreneurs dominating in openness, extroversion, conscientiousness, and agreeableness traits are more likely to lead and manage their small retail family firms to the next generation, but those with neuroticism trait may not. In addition, the mediating mechanism of *Descendant Entrepreneur Family Business Value Congruence (“DE-FBVC”)* revealed that the family firm’s innovative and sociable values activate the openness and extroversion traits of a descendant entrepreneur, which contribute to the success of the firm’s succession. In contrast, the firm’s risk-averse and irrational decision-making values diminish the neuroticism trait in a descendant entrepreneur’s initiatives toward succession success.

Our study offers three contributions. First, the present study contributes to the literature by examining the direct link between Big-5 personality traits and succession success of small family firms. In addition, this study investigates a novel mediating mechanism of DE-FBVC between the descendant entrepreneur’s Big-5 personality traits and the succession success of small family businesses. It is important to investigate this mediation because recent evidence in family business research suggests that the involvement of family members with different personality traits and conflicting values in the family firm create complex human capital management issues, especially for small family businesses (Gomez-Mejia et al., 2018). Second, we apply the person-organization fit theory to evaluate the descendant entrepreneur’s personality trait role theoretically and empirically in a small family business succession setting (Rauthmann, 2020). We utilize the mediating mechanism of DE-FBVC to explain the direct relationship between the Big-5 personality traits and succession success. Third, our results can assist retail sector business development consultants and predecessors in selecting the most appropriate descendant entrepreneur or delegating job responsibilities based on the descendant entrepreneur’s personality trait congruent with the business operations and values prevailing in small family businesses. Moreover, the attention of policymakers and family members toward the congruence of descendent entrepreneurs’ personality traits and values of small family businesses not only improves the likelihood of succession success but may also decrease the high rate of closing down of businesses due to succession failure.

## 2. Theoretical background and hypothesis development

### 2.1. Personality traits

Personality refers to a set of psychological characteristics that impact and explain persistent and distinct patterns of emotion, thoughts, and behaviors (Roberts et al., 2017). Researchers have studied personality theories for many years. These theories have suggested everything from 4,000 varieties of traits to Hans Eysenck’s three-factor theory. Through refinement, researchers mostly converged on the five-factor theory, also known as The Big-Five Personality Traits (Müller and Schwieren, 2020). The original five-factor model (big-5 personality traits) was proposed by Ernest Tupes and Raymond Christal in the early 1960’s. However, it did not reach widespread use until Lewis Goldberg’s work in lexical hypothesis emerged. After Goldberg’s work, other researchers, such Costa and McCrae, have used a similar research methodology of lexical hypothesis and reached similar groupings. Each researcher may have different names for their traits however, they all converge to the same set of big-5 personality traits.

Of all the personality characteristics, the Big-5 personality traits remains the most operationalized model of personality to date (Costa and McCrae, 1992; Canales García and Montiel Méndez, 2022). It consists of five personality traits, which are: *openness, conscientiousness, extroversion, agreeableness, and neuroticism*. Personality plays a pivotal role in the development of concepts of entrepreneurship such as the entrepreneurial career choice (Li et al., 2020; Batool et al., 2022), entrepreneurial cognitions (Cai et al., 2021), entrepreneurial motivation (Ismail, 2022), new venture survival (Chen et al., 2017), entrepreneur’s intentions (Farrukh et al., 2017), business performance (Hachana et al., 2018) and business life cycle (Zapalska and Wingrove-Haugland, 2018). Notably, Rauch and Frese (2007) examined specific personality traits such as achievement motivation and locus of control as determinants of an entrepreneur’s intention, strategies, and activities that lead to business success (Rauch and Frese, 2007). However, very little attention is paid to investigating the descendant entrepreneur’s personality traits on the success of family business succession.

### 2.2. Descendant entrepreneur-family business value congruence (“DE-FBVC”)

The concept of person-organization (descendent entrepreneur-family business) value congruence is derived from the person-organization fit theory. The person-organization value congruence is “a compatibility occurrence between an individual’s personality traits and firm’s values when their characteristics are well-matched” with each other (Kristof-Brown et al., 2005, p. 283). Lu et al. (2022) explain the person-organization value congruence as follows: “the congruence between patterns of organizational values and patterns of individual’s characteristics.” Similarly, Peng et al. (2015) define person-organization value congruence as “the congruence between the qualities of individuals (goals, skills, traits, behaviors, and attitude) and those of organizations (objectives, values, resources, and culture).” Therefore, the congruence of organizational values with the employee’s traits instigates his motivation and bolsters the firm’s success (Denison et al., 2014). For instance, an extrovert employee would work passionately in an organization whose values support him to conduct outdoor meetings with clients, but an introvert employee would not prefer to do so. In contrast, an extrovert employee might be demotivated if the values of the organization restrict him in building relationships with his clients or confine him to work in isolation (Rau et al., 2019).

Person-organization value congruence research follows the tradition of the attraction-selection-attrition paradigm (Abbasi et al., 2022), which argues that person-organizational congruence results from the similarity between the individual and the organization values (Pötschke, 2020). Thus, the congruence of organizational values with the employee’s traits instigates his motivation and bolsters the firm’s success (Denison et al., 2014). More specifically, a family firm’s values have a potent influence over the family business, as they contain long-term visions for the family firm’s performance and survival across generations (succession success) (Rau et al., 2019; Jiatong et al., 2021). Thus, the congruence of family business’s values with the descendant entrepreneur is an important predictor for succession success in the family business literature (Ahmad et al., 2022).

The person-organization value congruence concept has been widely accepted in human resource and organizational behavior research (Tabane et al., 2013). This concept derives that attracting and retaining individuals is easier if congruence exists between an individual’s traits and the organization’s values (Baruch and Rousseau, 2019). In recent years, researchers have observed that a mismatch of descendent entrepreneur’s personality traits with the family firm’s values creates problematic issues in human capital management for small family businesses (King et al., 2022). However, despite the complex and unresolved issues, studies on value congruence in family business research remain scarce to date (Pötschke, 2020).

Moreover, to the best of our knowledge (Hauswald et al., 2016), are the only authors to address person-organization value congruence in family firms. They revealed that job seekers with specific personality traits were found to be more attracted toward family firms having conservative values than self-enhancement values. Likewise, Rau et al. (2019) underlined the importance of values in family business research by explaining that family members’ involvement in management, ownership or governance influences the business values. Rau et al. (2019) further stressed that family members’ influence on the business values can be destructive or constructive, relying on their cognitive capabilities, which are linked with their personality traits.

Despite a large amount of general research on value congruence, there is a gap in the current person-organization-fit and family business literature in terms of descendent entrepreneur’s characteristics (personality traits) that might influence value congruence with the family firm (Abbasi et al., 2022). Thus, there is a strong need to empirically examine family firms’ values and personality traits of descendant entrepreneurs as potential determinants and mediators of the family firm succession (AlOmari, 2021). As such, this paper examines the relationship between a descendant entrepreneur’s personality traits and its congruence with the family business values to uncover how this potential relationship affects succession success.

### 2.3. Openness

Individuals who possess high openness trait like to try new things (Christensen et al., 2019). They explore and take on a broad range of interests and are very imaginative. They engage with others on a personal level because they are naturally curious about others (Connelly et al., 2014). An entrepreneur with a strong openness personality trait is said to be receptive to new experiences, creative, inventive, unorthodox, intellectually curious, and have a wide range of interests. An open entrepreneur is innovative and believes in ultimate diversity in network management and knowledge-sharing (Costa and McCrae, 1992; Udayanganie et al., 2019). Entrepreneurial literature indicates that an entrepreneur with an openness trait has creative behavior (Botero et al., 2021) and enjoys socializing and networking with existing and potential clients (Wolff and Kim, 2012). Thus, their positive work behavior (Zhou et al., 2022) results in high business performance. Similarly, it is envisaged that openness personality trait pertinent in a descendant entrepreneur of a family business would most likely ensure a successful succession transition. This is because the succession success of family firms requires adaptability in dynamic business scenarios, and these adaptive behaviors are outcomes of the openness trait.

An entrepreneur with an openness personality trait has the intellectual capacity to adapt to diverse situations (Baruch and Rousseau, 2019); and high intellectual stimulation values also drive him toward high achievement (Nieß and Zacher, 2015). According to Reina et al. (2014), the openness personality trait accelerates behavioral integration, inner harmony, and wisdom values. In turn, this will enhance the firm’s performance (Shashi et al., 2019). Further, Cohen et al. (2019) find that an entrepreneur with an openness trait is more motivated to manage businesses that share the same value in innovative approaches in business operations. Thus, it is predicted that a descendant entrepreneur’s openness trait which is congruent with the business values, will result in the success of the family business succession (Zhou et al., 2022). Hence, DE-FBVC promotes the openness trait of a descendant entrepreneur’s contribution to the success of the small family business succession.

*(H1a): Openness trait is positively related to the succession success of small family business.*

*(H1b): DE-FBVC mediates the relationship between the openness trait and succession success in the small family business.*

### 2.4. Conscientiousness

Individuals who are conscientious tend to be very thoughtful and intentional. In addition, they are goal-driven and organized (Roberts et al., 2017). An entrepreneur with conscientiousness personality trait is trustworthy, diligent, methodical, self-disciplined, and resourceful, with a strong drive for task accomplishments. Studies show that conscientiousness trait is a significant component in an entrepreneur’s work motivation (Huber et al., 2021). This finds support in a meta-analysis by Ellen et al. (2022), which reveals that an entrepreneur’s behavioral indicators (i.e., business resources utilization, business performance attainment commitment) are predicted by his conscientiousness trait. Since these behavioral indicators result in goal achievement motivation, the conscientiousness personality trait is found to have a strong relationship with elevated business performance (Jain and Verma, 2022). A business’s strong performance leads to business longevity and, thus, succession success. In sum, conscientiousness personality trait appears necessary to enhance succession success in small family businesses.

Franco and Prata (2019) observe that business values influence the performance of conscientiousness trait entrepreneurs. A conscientious entrepreneur would be more career-oriented when the firm’s conscientiousness related values (i.e., competence, achievement-striving, self-discipline, and deliberation) support him (Volmer et al., 2016). Furthermore, researchers have discovered that an organization’s achievement-focused values support the career advancement (Benson et al., 2020) and managerial advancement (Rabenu et al., 2021) of an entrepreneur with a conscientious personality trait. This indicated that a conscientiousness trait entrepreneur is more likely to accomplish specified set of goals (for example, sales targets achievement) in an achievement-oriented valued firm because of the congruence of personality trait with the values of the business. Thus, in the context of the small family business, the DE-FBVC generates an opportunity to establish competitive advantage and business success (Haseeb et al., 2019). Hence, the congruence of a conscientious descendant entrepreneur with their family business values may play an essential role in enhancing business longevity (de Groot et al., 2022). We argue that DE-FBVC intervenes in the relationship between the conscientiousness personality trait of the descendant entrepreneur and the succession success of small family businesses.

*(H2a): Conscientiousness trait is positively related with succession success of small family business.*

*(H2b): DE-FBVC mediates the relationship between the conscientiousness trait and succession success in the small family business.*

### 2.5. Extroversion

Individuals who score high on extroversion, or extroverts, are highly sociable and talkative (Diener and Lucas, 2019). They are very expressive with their emotions and may even seem assertive. They also recharge by being with friends. An entrepreneur who has dominant extroversion personality traits is sociable, conversational, affectionate, enthusiastic, person-oriented, and confident. Such characteristics are important to develop and maintain relationships with suppliers, consumers, and peers in a business (Canales García and Montiel Méndez, 2022). Further, entrepreneurs have to engage in negotiations with stakeholders, communicate with their workers, peers, siblings, and seek advice outside their social circles (Liang et al., 2015). Compared to an introvert, an extrovert entrepreneur may find such a task effortless. Reliance on social networks and business community support may be critical for overcoming entrepreneurial and succession success obstacles (Elo and Dana, 2019). Thus, an entrepreneur with a strong extrovert personality may lead the business toward subsequent generations more effectively. This is supported in the meta-analysis conducted by Pletzer et al. (2021), where the authors found that extroversion is a strong personality predictor of business performance.

Studies have revealed that a firm with risk-taking values improves the optimism (Aren and Nayman Hamamci, 2020) and confidence (Diputra and Arismunandar, 2021) of the entrepreneur, and this will facilitate him to control and lead the business successfully. Extroversion-related values, interactive communication, win-win negotiation roles, dynamic marketing tactics and adaptiveness ensures the extroversion trait leader’s business success due to congruence between the entrepreneur’s extroversion trait and extroversion related values of the firm (Cogliser et al., 2012). Thus, a descendant entrepreneur with extroversion personality trait which is congruent with the small family business values increases the likelihood of succession success.

*(H3a): Extroversion trait is positively related to the succession success of the small family business.*

*(H3b): DE-FBVC mediates the relationship between the extroversion trait and succession success of the small family business.*

### 2.6. Agreeableness

Agreeable individuals tend to hold attributes of trust and kindness (Ali et al., 2021). They are cooperative and are very helpful. They are usually caring and honest individuals. They have integrity, gentleness, helpfulness, naivety, compassion, and forgiveness attributes. Agreeableness trait also reflects interpersonal interactions, especially the inclination to be pleasing in a business context. For example, altruistic, empathic, thoughtful, and supportive entrepreneurs have high levels of agreeableness (John, 2021); all of such characteristics might be helpful when businesses are facing performance and sustainability challenges. Prior studies find that an entrepreneur who is dominant in agreeableness personality trait has a vision for business success (Teruel-Sánchez et al., 2021) and the allure of the business longevity (Honjo and Kato, 2022). Additionally, it has been demonstrated that the agreeableness personality trait positively correlates with business success because such an entrepreneur places importance in establishing internal networking values and culture (Coudounaris and Arvidsson, 2021). We, therefore, expect a positive relationship between the descendant entrepreneur’s agreeableness personality trait and the small family business’s succession success.

According to Cogliser et al. (2012), the performance of a service-focused firm may improve if its values, cooperation, friendliness, responsibility recognition, and kindness are consistent with the manager’s personality trait of agreeableness (Sun and Shang, 2019). Similarly, the agreeableness trait descendant entrepreneur is motivated in micro-enterprises having social care values (Patel and Thatcher, 2014). In this situation, the agreeableness trait descendant entrepreneur may preserve trustworthy relationships with their customers and stakeholders which is important for the successful succession transition (Deferne et al., 2022). Thus, the congruence of business values with the descendant entrepreneur’s agreeableness personality trait enhances the likelihood of succession success of the family business.

*(H4a): Agreeableness trait is positively related to succession success of the small family business.*

*(H4b): DE-FBVC mediates the relationship between the agreeableness trait and successions success of the small family business.*

### 2.7. Neuroticism

An entrepreneur’s tendency toward anxiety, depression, self-doubt and other negative feelings is termed neuroticism (Mamat et al., 2021). Individuals who score high on neuroticism tend to be highly stressed and moody. They are irritable and can even be anxious, making it difficult for them to remain calm in difficult situations. While this may seem negative, neurotic individuals tend to be more introspective. A neurotic entrepreneur exhibits a high level of negative emotions such as worry, distraction, despair, and helplessness (López-Núñez et al., 2020). Such an entrepreneur is more sensitive to stress and has less endurance and low emotional stability, particularly in a highly stress situation like during an economic recession. Thus, a neurotic entrepreneur will not perform well in analytical thinking situations or in a highly pressured job. Further, his tendency of being dissatisfied, ineffective, absent, or even leaving his job is very high (Adegbuyi et al., 2018). Such an entrepreneur may also exhibit poor job responsibilities in dealing with customers with different beliefs, cultural values, social orientation, and temperaments (Marsh et al., 2013). Meta-analyses reveal a negative correlation between neuroticism and business performance (Dimitriou and Galanakis, 2022). Therefore, it can be deduced that a descendant entrepreneur with a neuroticism personality trait may have a negative influence on the succession success of the small family business.

The neurotic entrepreneur’s work behavior is toxic to feedback and complex business settings (Zhao et al., 2021). A neurotic personality trait entrepreneur performs poorly in stress-oriented valued firms. In addition, research finds that neuroticism trait influence career success negatively (Martin et al., 2016). However, this negative influence may be neutralized where the organization avoids adversity and has security-assuring values (Nicolaou et al., 2021). Sinha and Srivastava (2013) also observed that entrepreneur with neuroticism trait is drawn to firms with low risk-taking values. Though such value congruence may motivate a neurotic employee to carry out his tasks, believing it is sufficient for the organization, he may still be skeptical of the organization’s success (Ahmed et al., 2022). Thus, though descendant entrepreneur-family business value congruence (DE-FBVC) may boost a neurotic individual’s drive, he may pose a threat to the firm’s long-term viability. Therefore, it is hypothesized that DE-FBVC mediates between the descendant entrepreneur’s neuroticism trait and succession success of small family businesses negatively.

*(H5a): Neuroticism trait is negatively related to the succession success of the small family business.*

*(H5b): DE-FBVC mediates the relationship between the neuroticism trait and succession success of small family businesses negatively.*

### 2.8. Conceptual framework


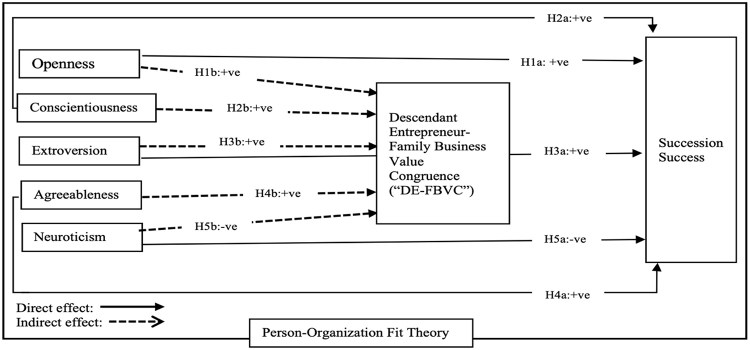


## 3. Methodology

The survey for this study was carried out using a pre-designed self-administered questionnaire based on prior personality traits and family firm research (Yusoff et al., 2021). We selected Pakistan’s small family businesses as our target population for two reasons. First, Pakistan is a developing country with economic and political instability (Mansoor et al., 2018). Second, the political and economic instability of a developing country such as Pakistan may result in a high likelihood of family business failure and thus, it is crucial to understand the factors that could lead to the successful succession of small family businesses despite the negativities. This study was carried out in four business hub cities namely Lahore, Faisalabad, Sialkot, and Gujranwala, all of which are situated in the most populous province, “Punjab” of Pakistan (Ahmad and Ahmad, 2021). These four cities were selected based on the following rationale: (1) Lahore is the largest trading city contributing 5.6% of Pakistan’s GDP; (2) Faisalabad is the largest industrial and distribution center connected with roads, rails, and air transportation; (3) Gujranwala contributes 5% of total GDP; and (4) Sialkot generates 10% of Pakistan’s overall exports through sports goods production. Moreover, these chosen business hub cities have a history dating back to Pakistan’s independence (August 1947), explaining why they have a higher family business concentration.

We obtained the list of SMEs from the Regional Business Centers of the four cities and selected our sample of small family firms based on the following criteria: (1) the business must be at least 5 years old (Faccio et al., 2016); (2) the business has 20–50 employees and an annual turnover of less than PKR 155 million (Jassim and Khawar, 2018); and (3) the business identifies itself as family-owned (Westhead and Cowling, 1998), and/or a single-family owns 50% or above shares (Chua et al., 1999). The lack of an accurate and updated sampling frame led us to use a non-probability purposive sampling (Isaga, 2015). We chose to examine the retail sector because it accounts for 18.2% of Pakistan’s GDP, and focusing on a single sector would offer better insight into that sector without complexities (Khan et al., 2020).

An expert panel comprising three experts in the field of the family business was established. In June 2021, the close-ended questionnaire after the content validation of experts was turned into a Google form and delivered to the target family businesses. Following the contact information provided by the Regional Business Centre of each of the four cities, 347 family businesses were contacted through email and text messages (WhatsApp). Brief information was provided about the nature of the study and the respondents were asked for their volunteer in participation. A list of 318 volunteer descendant entrepreneurs participants, designated as chairman and managing directors was prepared. Google form questionnaires were distributed through email and text messages (WhatsApp) and followed up after 2 weeks. The final sample consisted of 124 descendant entrepreneurs who responded, representing a response rate of 39%. Table 1 below shows the demographic information of the descendant entrepreneur respondents.

**TABLE 1:** Demographic information of respondents.

		Frequency	Percentage
Gender	Male	115	93%
	Female	9	7%
Designation	Chairman	77	62%
	Managing director	47	38%
Education level	Under-graduate	69	55%
	Graduate	41	33%
	Post-graduate	14	12%
Generation level	2nd generation	81	65%
	3rd generation	35	28%
	4th generation	8	7%

Authors’ own calculation.

### 3.1. Common method bias

We took precautions to avoid common method bias before data collection by implementing the following: (1) the research instrument included a cover letter and explicit approval form; (2) the objective of the study was described; (3) the respondents’ response was kept confidential; (4) the survey instrument was split into different sections; and (5) the variable “attitude toward the color blue” was employed as a marker variable (Cooper et al., 2020). In addition, Massey and Tourangeau (2013) indicated the likelihood of bias due to high non-responder rates. Therefore, this study analyzed the non-response bias as well.

### 3.2. Non-response bias

To assess the non-response bias, we took the following two steps. First, the data set was divided into two halves. In terms of sales and years in business, the first 25% and the last 25% of responses (i.e., two groups) were examined (Fricke et al., 2020). The results of the ANOVA test indicated that there was no significant difference between the two groups. Additionally, the Mann-Whitney *U*-test was used to compare the respondents and non-respondents of small family businesses and revealed non-significant findings. It is interesting that some non-respondents returned the survey with few sections of the questionnaire filled and justified their inability to respond by stating a lack of time. Considering these factors, it was found that non-response bias was minimal (Cheung et al., 2017).

### 3.3. Data analysis technique

We used the Smart-PLS software for three reasons. First, Smart-PLS software helps to overcome multicollinearity issues. Second, the model of this study had formative and reflective constructs, and third, our sample size was small (Hair et al., 2021). This study used a nomological approach to decide whether constructs are formative or reflective (Finn et al., 2014). Based on the nomological approach and literature support, this study treated personality traits and value congruence as reflective, while succession success was treated as a formative construct. This study used the PLS algorithm to test the validity and reliability while bootstrapping was used to analyze the relationship between the constructs (Sarstedt et al., 2021).

### 3.4. Variables

#### 3.4.1. Personality traits

The well-known Big-5 personality traits scale which is treated as a reflective construct and validated by John and Srivastava (1999). This scale has been widely utilized and validated in the setting of family businesses (Franco and Prata, 2019) and in the Asian context (Ayob and Makhbul, 2020). Furthermore, this scale has considerable empirical and theoretical overlap with Big-5 personality trait measures (Denissen et al., 2008). This measure was designed using a 5-point Likert scale spanning from: 1 (strongly disagree) to 5 (strongly agree). We adapted (John and Srivastava, 1999) Big-5 personality traits scale with five dimensions: *Openness, Conscientiousness, Extroversion, Agreeableness* and *Neuroticism*. Each question started with, “I see myself as someone who…..”

We measured *Openness* using the following 10 items, for example: “is original, comes up with new ideas,” “is curious about many different things,” “is ingenious, a deep thinker,” “has an active imagination,” “is inventive,” “values artistic, aesthetic experiences,” and “likes to reflect, play with ideas.” We measured *Conscientiousness* using nine items such as: “does a thorough job,” “is a reliable worker,” “perseveres until the task is finished,” “does things efficiently,” and “makes plans and follows through with them.” We measured *Extroversion* using eight items, for example: “is talkative,” “is full of energy,” “generates a lot of enthusiasm,” “has an assertive personality,” and “is outgoing, sociable.” We measured *Agreeableness* using nine items such as “is helpful and unselfish with others,” “has a forgiving nature,” “is generally trusting,” “is considerate and kind to almost everyone,” and “likes to cooperate with others.” Finally, we measured *Neuroticism* using eight items, for example: “is depressed,” “can be tense,” “worries a lot,” “can be moody,” and “gets nervous easily.”

#### 3.4.2. Value-congruence

Value congruence consisted of two scales: subjective-fit and objective-fit. The subjective fit is a match between the employee’s own values and his perception of organizational values. In contrast, objective-fit compares the employee’s values with the organizational values seen by other people (i.e., manager, co-workers). This study followed the concept of subjective fit (i.e., congruence of person-organization value) based on the respondent’s observation using the Person-Organization value congruence construct developed by previous studies (DeRue and Morgeson, 2007). We treated person-organization fit as a reflective construct with three items, adapted from Cable and DeRue (2002, p. 879): *“The things that I value in life are very similar to the things that my organization values”; “My personal values match my organization’s values and culture”;* and *“My organization’s values and culture provide a good fit with the things that I value in life.”* The reliability of this scale was (α = 0.860) in our sample, offering a close match to those validated by Cable and DeRue (2002), thus providing satisfactory internal consistent reliability. This 3-item scale was recorded using a 5-point Likert type ranging from 1 (strongly disagree) to 5 (strongly agree).

#### 3.4.3. Succession success

Similar to previous studies, this study treated succession success as a formative construct and validated it by using a 5-point Likert scale (Venter et al., 2005). Cabrera-Suárez and Martín-Santana (2012) succession success was measured using the following items: “*Relationship with suppliers, customers, financial institutions, etc., have not been damaged by the change of management”; “The expectations for the future of the firm are favourable”; “The firm has improved its strength and competitive position since I have been working in it”; “The working atmosphere and employee satisfaction have improved”; “The family is satisfied with the evolution of the firm”; “I am satisfied professionally with the evolution of the succession process.”*

## 4. Results

### 4.1. Reliability and validity

We examined our model using the PLS-SEM statistical approach, utilizing Smart PLS 3.3.3 statistic software to test the reliability and validity (Hair et al., 2021). Table 2 shows the reliability and validity of all our studied variables. All variables surpass the recommended threshold of 0.708, with Cronbach’s alpha exceeding 0.7 (Hair et al., 2019). The average variance extracted (AVE) also exceeds 0.5, indicating reliability and convergent validity (Purwanto and Sudargini, 2021).

**TABLE 2:** Exogenous construct’s reliability and validity.

		Convergent validity				Internal consistency	Reliability
		Outer loading	Indicator reliability	*t*-value	AVE	Composite reliability	Cronbach’s α
Constructs	Indicators	>0.7	>0.5	>2.	>0.5	>0.7	>0.7
Agreeableness	AG.1	0.824	0.000	33.103	0.695	0.954	0.946
	AG.2	0.821	0.000	28.925			
	AG.3	0.870	0.000	70.965			
	AG.4	0.876	0.000	76.184			
	AG.5	0.865	0.000	62.227			
	AG.6	0.781	0.000	29.759			
	AG.7	0.780	0.000	26.298			
	AG.8	0.885	0.000	50.325			
	AG.9	0.798	0.000	34.579			
Conscientiousness	CO.1	0.836	0.000	34.050	0.670	0.948	0.938
	CO.2	0.834	0.000	37.962			
	CO.3	0.875	0.000	45.394			
	CO.4	0.834	0.000	40.392			
	CO.5	0.793	0.000	22.827			
	CO.6	0.796	0.000	28.751			
	CO.7	0.842	0.000	37.852			
	CO.8	0.821	0.000	29.709			
	CO.9	0.725	0.000	19.107			
Extroversion	EX.1	0.609	0.000	14.217	0.541	0.904	0.879
	EX.2	0.739	0.000	22.473			
	EX.3	0.722	0.000	22.749			
	EX.4	0.817	0.000	38.023			
	EX.5	0.752	0.000	23.849			
	EX.6	0.762	0.000	22.682			
	EX.7	0.763	0.000	42.776			
	EX.8	0.701	0.000	22.327			
Neuroticism	NE.1	0.790	0.000	30.009	0.501	0.888	0.862
	NE.2	0.782	0.000	26.125			
	NE.3	0.617	0.000	13.055			
	NE.4	0.739	0.000	18.753			
	NE.5	0.708	0.000	23.153			
	NE.6	0.749	0.000	23.629			
	NE.7	0.636	0.000	11.561			
	NE.8	0.605	0.000	10.419			
Openness	OP.1	0.794	0.000	34.0804	0.539	0.920	0.906
	OP.2	0.801	0.000	33.624			
	OP.3	0.557	0.000	12.670			
	OP.4	0.708	0.000	28.326			
	OP.5	0.756	0.000	26.367			
	OP.6	0.762	0.000	24.982			
	OP.7	0.714	0.000	19.309			
	OP.8	0.666	0.000	16.235			
	OP.9	0.755	0.000	20.682			
	OP.10	0.791	0.000	42.451			
Descendent entrepreneur-family business value congruence	DE-FBVC.1	0.819	0.000	35.601	0.634	0.838	0.723
	DE-FBVC.2	0.733	0.000	12.119			
	DE-FBVC.3	0.833	0.000	47.027			

Authors’ own calculation.

Pertaining to an endogenous construct’s reliability and validity, Table 3 shows that the indicators of the succession success construct have no collinearity issues since the VIF value for each indicator is less than five (Sarstedt et al., 2021).

**TABLE 3:** Endogenous construct’s reliability and validity.

		Significance and outer weight				Collinearity	Convergent validity
			*t*-value	Outer loading	*t*-value	VIF	Redundancy analysis
FBP	Indicators	Outer weight	>1.96	>0.5	>1.96	<5	>0.7
Succession success	RCM	0.168	4.629	0.516	7.881	1.440	0.760
	EFF	0.293	14.855	0.887	67.569	3.110	
	ICP	0.205	8.603	0.756	16.343	2.191	
	ASI	0.272	11.255	0.831	35.238	2.241	
	SE	0.249	7.803	0.696	13.627	1.773	
	SESP	0.203	6.376	0.478	6.549	1.318	

Authors’ own calculation.

Table 4 shows the results to estimate discriminant validity. All relevant variables are reliable and valid for testing purposes using PLS-SEM.

**TABLE 4:** Discriminant validity.

Constructs	AG	CO	EX	NE	OP	DE-FBVC	SS
	AVE = 0.696	AVE = 0.670	AVE = 0.541	AVE = 0.500	AVE = 0.539	AVE = 0.633	Formative
AG							
CO	0.799						
EX	0.521	0.483					
NE	0.523	0.613	0.521				
OP	0.609	0.620	0.598	0.703			
DE-FBVC	0.737	0.707	0.584	0.441	0.560		
SS	0.391	0.346	0.568	0.352	0.620	**0.796**	0.485

Authors’ own calculation.

The structure model was examined using bootstrapping through Smart PLS 3.3.3 software to obtain statistical results and verify our research assumptions (Ringle et al., 2015). The findings provide evidence to establish ranges of confidence for parameter accuracy evaluation. Table 4 depicts the predictive relevance of the structural model. It can be observed that the descendant entrepreneur’s personality traits influence the DE-FBVC by 47.6% (*R*^2^= 0.476), and succession success by 23.5% (*R*^2^= 0.235). This results show that the model is of quality, and the outcomes are beneficial for business decision-making (Hair et al., 2017).

### 4.2. Direct effect

Table 5 is termed “direct path analysis,” indicating the direct association between Big-5 personality traits and succession success. The results regarding H1a, on the relationship between openness and succession success (β = 0.207, *t* < 2.695) revealed that openness traits have a significant positive association with succession success. Thus, H1a is supported. It has been observed that the openness trait of the descendant entrepreneur has a 20.7% influence on the success of a small family business’s succession. Referring to the test by Cohen et al. (2019), the f^2^ = 0.048 magnitude is small in size. As such, it can be inferred that openness trait descendant entrepreneurs have the capability of 4.8% to bring about the successful succession of their small family businesses.

**TABLE 5:** Direct path analysis.

Hypothesis	Path	Co-efficient	*t*-values	Supported	f^2^	*R* ^2^
(H1a)	OP → SS	0.207	2.695	Accepted	0.048	0.739
(H2a)	CO → SS	0.415	7.809	Accepted	0.195	
(H3a)	EX → SS	0.311	6.892	Accepted	0.207	
(H4a)	AG →SS	0.233	3.810	Accepted	0.066	
(H5a)	NE → SS	-0.045	0.856	Rejected	0.003	

Next, the results regarding H2a, on the relationship between conscientiousness and succession success (β = 0.415, *t* < 7.809) indicate that the conscientiousness trait positively impacts the succession success of small family businesses. Therefore, H2a is also supported. In fact, the conscientiousness trait of the descendant entrepreneur impacts 41.5% of the succession of small family businesses and the magnitude of f^2^ = 0.195 is medium in size. This show that a conscientiousness trait descendant entrepreneur may secure 19.5% likelihood for successful succession (Cohen et al., 2019).

The results in Table 5 relating to H3a, on the relationship between extroversion and succession success (β = 0.311, *t* < 6.892) shows that the extroversion trait significantly influences the succession success of small family businesses. Hence, H3a is also supported. The extroversion trait of the descendant entrepreneur has a 31.1% influence on the success of small family businesses’ succession and the f^2^ = 0.207 magnitude is medium-sized (Cohen et al., 2019). Hence, it can be inferred that extroversion trait descendant entrepreneurs may contribute 20.7% to the successful succession of their small family businesses.

The findings for H4a on the relationship between agreeable and succession success (β = 0.233, *t* < 3.810) shows that the agreeableness trait positively influence the succession success of small family businesses. Therefore, H4a is supported. Results show that the descendant entrepreneur’s agreeableness trait impacts 23.3% of the succession success of small family businesses. Referring to the Cohen et al. (2019) test, f^2^ = 0.066 is small in magnitude, indicating that the agreeableness trait of a descendant entrepreneur contributes low in the succession success of small family businesses. It can be inferred that the agreeableness trait of a descendant entrepreneur contributes about 6.6% to the succession success of their small family businesses.

With regards to H5a, the results on the relationship between neuroticism and successful succession (β = −0.045, *t* > 0.856) reveal that neuroticism trait descendant entrepreneurs may not have the capabilities for the successful succession of small family businesses. Thus, H5a is not supported.

Taken together, we can deduce that the descendant entrepreneur’s conscientiousness trait is ranked first in contributing to the successful succession of small family businesses, while extroversion ranked second, agreeableness ranked third, and openness ranked fourth. However, a descendant entrepreneur with neuroticism trait does not contribute to the successful succession of the small family business.

### 4.3. Mediating effect

Next, we investigate whether DE-FBVC functions as a mediator in the association between the descendant entrepreneur’s Big-5 personality traits and succession success. The mediation analysis was conducted in a similar manner as past studies (Hair et al., 2017). The findings are shown in Table 6. The mediating effect of DE-FBVC between openness trait and succession success is significant (H1b: β = 0.295, *t* = 7.477). The descendant entrepreneur’s openness trait is associated with succession success, and DE-FBVC also mediates between the aforesaid relationship. Thus, there is partial mediation and H1b is supported. Since the partial mediation sign is positive, we can infer a complementary mediation (i.e., partial mediation) (Zhao et al., 2010). To investigate the strength of complementary mediation, we perform the variance accounted for (“VAF”) by using the VAF formula of complementary mediation (Hair et al., 2017). This formula assesses how much of the variation in the dependent variable is accounted for directly by the independent factors and how much is accounted indirectly through the mediator variable (Zhao et al., 2010). The VAF is computed using the formula below:

**TABLE 6:** Indirect path analysis.

Hypothesis	Path	Co-efficient	*t*-values	Supported
(H1b)	OP → DE-FBVC → SS	0.295	7.477	Accepted
(H2b)	CO → DE-FBVC → SS	-0.036	0.989	Rejected
(H3b)	EX → DE-FBVC → SS	0.169	5.583	Accepted
(H4b)	AG → DE-FBVC → SS	0.008	0.216	Rejected
(H5b)	NE → DE-FBVC → SS	-0.110	3.325	Accepted


$$\text{VAF}=\frac{I\,n\,d\,i\,r\,e\,c\,t\,E\,f\,f\,e\,c\,t}{T\,o\,t\,a\,l\,E\,f\,f\,e\,c\,t}=\frac{\left(0.632*0.214\right)}{\left(0.632*0.214+0.295\right)}=0.314$$

Relying on the VAF outcomes, it is possible to deduce that the DE-FBVC is within the current study model. The mediation effect of the DE-FBVC explains 31.4% of the influence of the openness trait on succession success. Since this VAF is more than 20% but less than 80%, this condition may be classified as a partial mediation (Hair et al., 2017). On the other hand, the mediating effect of DE-FBVC between conscientiousness trait and succession success is not significant (H2b: β = −0.036, *t* = 0.989). Thus, although the conscientiousness trait has a significant direct relationship with succession success, the DE-FBVC does not mediate between the descendant entrepreneur’s conscientiousness traits and succession success. Thus, only a direct relationship is found (Ramayah et al., 2018) and H2b is not supported.

Nevertheless, the extroversion trait has a significant direct relationship with succession success, and DE-FBVC is also found to mediate between the relationship. The mediating effect of DE-FBVC between extroversion trait and succession success is significant (H3b: β = 0.169, *t* = 5.583). Thus, partial mediation prevails, supporting H3b. As the sign of the partial mediation is positive, we can infer a complementary mediation (i.e., partial mediation) between extroversion and succession success (Zhao et al., 2010). Since the implementation of VAF is required in the case of complementary mediation, we compute VAF using the formula below:


$$\text{VAF}=\frac{I\,n\,d\,i\,r\,e\,c\,t\,E\,f\,f\,e\,c\,t}{T\,o\,t\,a\,l\,E\,f\,f\,e\,c\,t}=\frac{\left(0.349*0.485\right)}{\left(0.349*0.485+0.169\right)}=0.500$$

The computation reveals that the DE-FBVC explains 50% of the influence of the extroversion trait on succession success. Since this VAF is more than 20% but less than 80%, this condition may be classified as a partial mediation (Hair et al., 2017).

The mediation analysis results reveal that the mediating effect of DE-FBVC between agreeableness trait and succession success is not significant (H4b: β = 0.008, *t* = 0.216). However, since the agreeableness trait has a significant direct association with succession success, there exists only a direct relationship and H4b is not supported.

Finally, we have earlier found that the neuroticism trait has no significant direct relationship with succession success. However, our analysis on the mediating effect of DE-FBVC between neuroticism trait and succession success shows significant results (H5b: β = −0.110, *t* = 3.325). Since DE-FBVC mediates the relationship between descendant entrepreneurs’ neuroticism trait and succession success despite having no direct relationship, full mediation prevails (Zhao et al., 2010). Thus, H5b is supported.

## 5. Discussion

This study investigates the direct effect between the descendant entrepreneur’s Big-5 personality traits and the succession success of the small family business (H1a to H5a) and the indirect effect through mediating mechanism of DE-FBVC between the Big-5 personality traits and succession success (H1b to H5b). Our results reveal that openness, conscientiousness, extroversion, and agreeableness personality traits of a descendant entrepreneur have a significant positive relationship with succession success. However, the neuroticism trait has no significant relationship with succession success. Therefore, our findings indicate that small family businesses with descendant entrepreneurs having specific traits of openness, conscientiousness, extroversion, and agreeableness enjoy higher rates of successful succession.

With regards to the mediating mechanism, DE-FBVC mediates the positive relationship between two personality traits: openness and extroversion, with succession success. Our results also show that DE-FBVC mediates the negative relationship between neuroticism and succession success.

Our results also suggest that a descendant entrepreneur with openness trait (i.e., intellectually curious, inventive, unorthodox) may contribute more toward the successful succession of small family businesses (H1a is accepted). The results of hypothesis H1a is consistent with the findings of Rashid et al. (2020), Botero et al. (2021), and Zhou et al. (2022). Botero et al. (2021) revealed that the openness personality trait has a significant relationship with job performance. Zhou et al. (2022) found that openness trait entrepreneurs have a significant association with financial and business growth performance and the success of small and local businesses (Rashid et al., 2020). Retail small family businesses expanding their operation’s diversity and introducing innovative products in a developing country like Pakistan may increase their profitability and expand customer segments. Thus, the openness trait of descendant entrepreneurs affiliated with these types of small family businesses would encourage innovation and creativity.

Our study revealed that the descendant entrepreneur’s conscientiousness trait has a significant positive association with the succession success of small family businesses (H2a is accepted). The outcome of H2a is in line with the findings of Judge et al. (2002), Ellen et al. (2022), and Jain and Verma (2022). For instance Judge et al. (2002), found that conscientiousness trait entrepreneurs have high job satisfaction because they are prone to perform tasks carefully for the firm’s success. Furthermore, Ellen et al. (2022) revealed that conscientious investors have a high proclivity for acquiring financial knowledge, making them less prone to financial investment loss. In addition, Jain and Verma (2022) indicated that conscientiousness trait project managers are more likely to accomplish their assigned projects. Since small family retail businesses are less hierarchical, descendant entrepreneurs who are conscientious are able to observe their firms’ affairs and performance very closely. This allows conscientious descendant entrepreneurs to contribute significantly to the succession success of their small family businesses.

We also found that descendant entrepreneur’s extrovert and agreeable traits have significant positive associations with succession success (H3a and H4a are accepted). The results of H3a is consistent with the work of Liang et al. (2015) and Manzano-García and Ayala-Calvo (2020). They emphasized the importance of developing relationships with suppliers and consumers to ensure the longevity of a business. An extrovert descendant entrepreneur would be able to handle networking with stakeholders and are likely to promote establishing strong relationships with clients, suppliers, and other stakeholders more frequently. The results of H4a are consistent with the findings of John (2021) and Teruel-Sánchez et al. (2021). They revealed that individuals with agreeableness traits accomplish the responsibilities assigned by their organization and achieve high success. In a similar vein, a descendant entrepreneur with agreeableness trait is cooperative, friendly, empathetic, and responsible, which will help preserve trustworthy relationships with their customers and stakeholders, critical for the successful succession transition and sustainability of the family business. As a result, extrovert and agreeable descendant entrepreneurs earn more sustainable benefits, better profits, and secure higher business performance through their relationships with stakeholders. Hence subsequently, they are more likely to contribute significantly, either directly or indirectly, to the succession success of their small family business.

We find that the neuroticism trait has no association with succession success (H5a is rejected). The results of H5a are consistent with Marsh et al. (2013), who revealed that a neuroticism trait entrepreneur has poor performance due to low analytical thinking. Thus, a highly neurotic descendant entrepreneur cannot foresee the future of their small family businesses and thus, may not contribute toward the success of its succession.

The results of H1b show that DE-FBVC mediates the relationship between openness and succession success (H1b is accepted). The results of H1b is consistent with the literature as openness trait CEOs nurture behavioral integration (Reina et al., 2014), innovative harmony, and creativity-supporting values, which accelerate the firm’s performance (Shashi et al., 2019). In addition, Reina et al. (2014) stressed that entrepreneurs with openness are more motivated to manage such businesses whose values are to opt for innovative approaches in their business operations. Thus, an entrepreneur’s openness trait congruent with innovation-related business values results in business success (Cohen et al., 2019) and subsequently, succession success. Hence, openness trait descendant entrepreneurs perform innovative tasks and gain competitive advantage when values and traditions support them, thus are likely to be more successful in leading their family business succession. For instance, innovation promulgating values of retail small family businesses support the openness trait of descendant entrepreneurs to expand it to the national level or permit creative operational diversification adoption to attain high business performance. Therefore, such innovation-supporting values which are congruent with the openness trait of a descendant entrepreneur may enhance the likelihood of succession success of small family businesses.

The results of (H2b) show that DE-FBVC reveals no mediating relationship between conscientiousness trait and succession success (H2b is rejected). The findings of H2b are in line with previous literature (Volmer et al., 2016; Rovelli et al., 2022). These authors found that founders or predecessors establish the values and traditions of small family businesses because they have greater experience, affiliation, and concern about their business progress. However, they underestimate their descendant entrepreneurs’ competency, achievements, and discipline toward their businesses. Thus, the descendant entrepreneur either gets little support or no support, which may not activate his or her conscientiousness trait to contribute toward the succession success of their small family business.

The mediating mechanism of DE-FBVCs significantly intervenes between extroversion trait and succession success positively (H3b is accepted). Previous studies endorse the findings of H3b and revealed that individuals having extroversion traits confront challenges and are ambitious to achieve goals in firms whose values support building social network (Diputra and Arismunandar, 2021). The extrovert individual with the support of extroversion-related values, utilize his relationship to earn profits and such congruence increases the likelihood of a firm’s success (Cogliser et al., 2012). Thus, small family business values promulgating interactive communication, negotiations, and social networking instigate the extroversion trait of descendant entrepreneurs to work more deliberately for performance and subsequently, succession success of the family firm.

The mediating results of DE-FBVC also reveal no mediating effect on the relationship between the personality trait of agreeableness and succession success (H4b is rejected). This results of H4b is in line with Patel and Thatcher (2014). Patel and Thatcher (2014) revealed that family firms have values to protect their interests, goal achievement, and attain competitive advantage. However, the agreeableness trait descendant entrepreneur is humble, kind, and selfless (Roccas et al., 2002). Therefore, in such a situation, the agreeableness trait descendant entrepreneur might not get the support of his small family business’s values and may result in succession failure.

The DE-FBVC significantly mediates the relationship between neuroticism and succession success negatively (H5b is accepted). Previous studies support the results of H5b and found that the prevalence of a firm’s risk-taking values instigates the low neuroticism trait (emotional stability) descendant entrepreneurs to lead in difficult and stressful circumstances (Ahmed et al., 2022), nourishing them to become more vigilant and experienced during recessions. This arrangement, which occurs as a result of the congruence of risk-taking values with low neuroticism traits, leads to the descendant entrepreneur’s contribution toward the succession success of the small family business. In contrast, a high neuroticism trait descendant entrepreneur may perform well in risk-averse valued small family businesses, but rapidly changing technological and marketing tactics will not provide enough space for risk-averse valued small family businesses to survive across generations, thus resulting in the failure of succession.

## 6. Theoretical implications

The findings of our study offer several theoretical implications. First, we contribute to the family business literature by showing that descendant entrepreneurs’ personality traits improve the likelihood of succession success of small family businesses. We found that a descendant entrepreneur who is open, conscientious, extrovert and agreeable is more likely to successfully lead his small family business into the next generation compared to a descendant entrepreneur who is neurotic. Second, drawing from the person-organization fit theory, the alignment of values of the small family business with the openness and extroversion traits of descendent entrepreneurs may result in a successful succession transition into subsequent generations. On the other hand, the small family business’s having neuroticism-related values similar to the neuroticism trait descendent entrepreneurs may facilitate him in avoiding bold decision-making and risk-taking. Such a situation may not only reduce the profit-earning opportunities, but also reduces the longevity of the neuroticism-valued family firm. For example, we find that if a family firm is perceived to have values associated with neuroticism such as vulnerability, insecurity in stakeholders’ relationships, risk-averse, coupled with a descendant entrepreneur who is neurotic, who can become anxious, tense and guilt-prone, then this descendant entrepreneur will most likely fail to lead the family firm into the next generation successfully. However, agreeableness and conscientiousness-related values of a small family firm do not support the agreeableness and conscientiousness trait descendent entrepreneur for successful succession. Third, our research design offers some initial evidence of the importance of DE-FBVC as a mediating mechanism that bridges the descendant entrepreneur’s Big-5 personality traits and the succession success of small family businesses.

## 7. Practical implications

Our study provides some initial evidence that a descendant entrepreneur’s personality traits play a role in the succession of family firms across generations. The findings of our study suggest that predecessors should select descendant entrepreneurs whose personality traits are aligned with the predecessor’s (or founder’s) values embedded within the family firm. Moreover, based on the person-organization fit theory, the predecessor should assign job responsibilities to their descendant entrepreneurs similar to their personality traits. Such practice will increase the descendant entrepreneur’s motivation to work more deliberately and nurture the values of that family firm. Therefore, a balanced combination of assigned job responsibilities with the descendant entrepreneur’s personality traits and values of the family firm may result in succession success. For example, a descendant entrepreneur who is an introvert may not be able to maintain a retail family business founded by his extrovert father because he is unable to maintain customer-building relationships and other social tasks that are essential to achieve competitive advantage and stellar performance in the family’s retail business. Thus, our findings provide some empirical support for the person-organization fit theory within the context of small family businesses.

## 8. Limitations and research directions

Even though our study’s objectives were met, several limitations must be considered. First, this research concentrates on small family firms in Pakistan’s retail industry, which may have limited applicability to other industries or states. Further research is needed to generalize results in other industries (e.g., manufacturing, tourism, health care, etc.) and advanced countries (e.g., China, USA). Second, the current study used a cross-sectional method to gather data which may result in common method variance (CMV). However, we assessed the CMV and found that this was not a problem. Third, future studies could utilize longitudinal research with a longer observation timeframe in order to examine how outcomes change over time. Fourth, while our focus is on small family businesses, future research should include large family businesses because small businesses may have different levels of resources, environment, or organizational hierarchy. Fifth, future studies can incorporate other personality traits (e.g., entrepreneurial, proactive, dark, and hexaco traits) for successful succession transition through the value congruence mediating mechanism. Finally, it will be interesting if certain moderators (e.g., relationship quality, trust, education) and mediators (e.g., entrepreneur’s commitment, motivation) are incorporated into the current model, adding to the business succession literature and extending the person-organization fit theory.

## 9. Conclusion

The present study provides some initial evidence that the alarmingly low succession success rate in small family firms might be due to the descendant entrepreneur’s personality traits and the congruence of the descendant entrepreneur’s personality traits with the values of their small family businesses. The results of our study suggest that descendant entrepreneurs with openness, conscientiousness, extroversion, and agreeableness traits possess the capabilities to lead their small family business into subsequent generations (succession success). However, due to their poor cognitive abilities, limited vision, and risk aversion characteristics, neuroticism trait descendent entrepreneurs may not be able to lead their small family business into subsequent generations.

Openness trait descendant entrepreneurs are creative, while extroversion trait descendant entrepreneurs believe in building relations with their colleagues and peers (Ogunfowora et al., 2022). Our study finds that the likelihood of successful succession increases when the values of small family businesses support the openness trait descendant entrepreneurs to perform innovative tasks, and the extroversion trait descendant entrepreneurs’ abilities to engage in social activities. On the other hand, despite having congruence with the values of their small family business, a descendant entrepreneur who is conscientious and agreeable may not be able to lead their family business into subsequent generations.

Despite having risk-averse (similar to neuroticism trait) or risk-taking (opposite to neuroticism trait) values, neuroticism-trait descendant entrepreneurs may be unable to pass on their small family business to the next generation. Family businesses with risk-averse values often do not last over the long term because of the unwillingness to take chances, at the expense of the firm’s profitability and longevity (Canales García and Montiel Méndez, 2022). Therefore, a descendant entrepreneur with neuroticism trait may find comfort in a risk-averse small family business, which can lead to succession failure. Moreover, a descendant entrepreneur high in neuroticism, may find it difficult to succeed in a risk-taking/opportunity-seeking firm due to his inability to evaluate the risks or opportunities effectively. In addition, a descendant entrepreneur with neuroticism trait may make irrational decisions or misjudge risks, leading to succession failure.

In conclusion, the descendant entrepreneur’s personality traits and the congruence of small family business values with the descendant entrepreneur’s personality traits may be required for succession success.

## Data availability statement

The original contributions presented in this study are included in the article/supplementary material, further inquiries can be directed to the corresponding author.

## Ethics statement

The studies involving human participants were reviewed and approved by the University of Malaya. The patients/participants provided their written informed consent to participate in this study.

## Author contributions

All authors listed have made a substantial, direct, and intellectual contribution to the work, and approved it for publication.

## Footnotes

1 For ease of reference, we refer to the descendant of an entrepreneur who takes over the control of the small family business started by the entrepreneur as “descendant entrepreneur”.
