# Corrigendum: Does congruence between a descendant entrepreneur's personality traits and family business values matter for succession?

**DOI:** 10.3389/fpsyg.2023.1181939

**Published:** 2023-03-24

**Authors:** Zeshan Ahmad, Wai Meng Chan, Elaine Yen Nee Oon

**Affiliations:** Faculty of Business and Economics, University of Malaya, Kuala Lumpur, Malaysia

**Keywords:** big-5 personality traits, family business value congruence, succession success, small family business, person-organization fit theory

In the published article, there was an error in the Funding statement as published. The correct Funding statement appears below.

